# Artificial Intelligence–Based Prediction of Subjective Refraction and Clinical Determinants of Prediction Error

**DOI:** 10.3390/diagnostics16020331

**Published:** 2026-01-20

**Authors:** Ozlem Candan, Irem Saglam, Gozde Orman, Nurten Unlu, Ayşe Burcu, Yusuf Candan

**Affiliations:** 1 Department of Ophthalmology, Ankara Training and Research Hospital, University of Health Sciences, 06340 Ankara, Türkiye; irem.saglam.458@gmail.com (I.S.); gozdeerkan@hotmail.com (G.O.); nurtenunlu@gmail.com (N.U.); anurozler@yahoo.com.tr (A.B.); 2 Energy Market Regulation Authority, 06510 Ankara, Türkiye; ycandan@epdk.gov.tr

**Keywords:** artificial intelligence, subjective refraction, machine learning, autorefractor, refractive error, prediction accuracy, ophthalmology

## Abstract

**Background/Objectives:** Subjective refraction is the clinical gold standard but is time-consuming and examiner-dependent. Most artificial intelligence (AI)-based approaches rely on specialized imaging or biometric data not routinely available. This study aimed to predict subjective refraction using only routine, non-cycloplegic autorefraction and keratometric data and to identify factors associated with reduced prediction accuracy. **Methods**: This retrospective study included 1856 eyes from 1006 patients. A multi-output histogram gradient-boosting model predicted subjective spherical equivalent, cylindrical power, and astigmatic axis. Performance was evaluated on an independent test dataset using R^2^ and mean absolute error, with circular statistics for axis prediction. Prediction failure was assessed using clinically relevant tolerance thresholds (sphere/cylinder ≤ 0.50 D; axis ≤ 10°) and multivariable logistic regression. **Results:** The model achieved high accuracy for spherical and cylindrical prediction (R^2^ = 0.987 and 0.933; MAE = 0.126 D and 0.137 D). Astigmatic axis prediction demonstrated strong circular agreement (ρ = 0.898), with a mean absolute angular error of 4.65° (median, 0.96°). Axis errors were higher in eyes with low cylinder magnitude (<0.75 D) and oblique astigmatism. In multivariable analysis, steeper keratometry (K2; OR = 7.25, 95% CI 1.62–32.46, *p* = 0.010) and greater objective cylindrical power (OR = 2.79, 95% CI 1.87–8.94, *p* = 0.032) were independently associated with poor prediction. **Conclusions:** A machine-learning model based solely on routine, non-cycloplegic autorefractor and keratometric measurements can accurately estimate subjective refraction, supporting AI as a complementary decision-support tool rather than a replacement for conventional subjective refraction.

## 1. Introduction

Refractive errors are among the most prevalent causes of correctable visual impairment worldwide. They affect over two billion individuals, particularly those in younger populations [1]. Although subjective refraction remains the clinical gold standard for prescription determination, it is a time-consuming process that is examiner-dependent and less feasible in pediatric and communication-limited groups, as well as in high-volume care settings [2]. Objective techniques, such as autorefraction, provide faster and more standardized measurements; however, the agreement with subjective refraction varies depending on age, accommodative status, and optical media quality. Consequently, clinical refinement is often required [3,4].

The use of artificial intelligence (AI) and machine learning (ML) methods in the assessment of refractive errors has increased substantially in recent years. These approaches can be broadly categorized into three groups. They include models that leverage routine tabular clinical data (e.g., biometric or optical measurements), deep learning systems trained on ocular images, and multimodal fusion frameworks that integrate complementary sources of information. Multimodal data fusion has been a subject of extensive exploration in the broader deep learning literature. This general strategy combines heterogeneous inputs to improve predictive robustness and generalizability across complex biomedical applications [5]. Machine learning (ML) models that incorporate biometric, optical, and demographic features have exhibited superior accuracy in comparison with conventional regression or autorefractor-only estimates, particularly in adult and pediatric populations [6,7,8]. Concurrently, the employment of deep learning models that have been trained on retinal fundus or ultra-widefield images has led to the capacity for direct prediction of spherical equivalent and detection of high myopia, thereby underscoring the potential of image-based inference [9,10,11].

In recent developments, multimodal fusion strategies have been investigated to expand predictions to encompass full refractive components, including sphere, cylinder, and axis, by integrating heterogeneous data sources such as ocular images, biometric parameters, and clinical measurements [9,12]. Furthermore, machine learning (ML)-based forecasting models have exhibited efficacy in predicting myopia progression and identifying individuals at risk of high myopia, thereby supporting risk-based follow-up strategies [13,14,15]. Systematic reviews and bibliometric analyses underscore the rapid expansion of AI applications in refractive error management, while also emphasizing persistent challenges related to external generalizability, device dependence, and heterogeneous performance across populations and settings [10,16,17].

Despite this progress, several limitations remain. A substantial proportion of published models depend on wavefront aberrometry, advanced ocular imaging, or large-scale image datasets, which may not be readily available in routine ophthalmology clinics or primary care settings [7,9,10,11,12,16]. Furthermore, a paucity of studies has systematically examined clinical determinants of prediction failure, such as near-emmetropic refractive status, age-related optical changes, or non-cycloplegic measurement conditions, that may critically influence accuracy in real-world settings [8,17]. Consequently, the role of AI-based refractive estimation in safely supporting clinical decision-making or patient triage without full subjective refinement remains incompletely defined.

The present study aims to address these gaps by developing a machine-learning model that predicts subjective refraction using routinely available, non-cycloplegic autorefraction and keratometric data, without reliance on specialized imaging or biometric equipment. In addition to evaluating the overall predictive performance, we specifically investigate clinical factors associated with reduced prediction accuracy. This enables us to identify the conditions under which artificial intelligence-assisted refraction may be considered reliable as a decision-support tool. This approach is intended to facilitate the safe and pragmatic integration of AI-based refraction into standard ophthalmic practice, particularly in high-volume clinical settings.

## 2. Materials and Methods

This retrospective cross-sectional study was conducted at a tertiary referral ophthalmology center between 1 January 2023 and 1 January 2025. The study was conducted in accordance with the tenets of the Declaration of Helsinki and received approval from the institutional ethics committee (Approval No. E-25-431, 16 April 2025). Written informed consent was obtained from all adult participants and from the legal guardians of minors. Prior to analysis, all data were de-identified to ensure patient confidentiality.

### 2.1. Study Population and Data Collection

The electronic medical records of 1006 consecutive patients were systematically reviewed. Eligible participants were required to be ≥7 years old, literate, and sufficiently cooperative for subjective refraction. Best-corrected visual acuity (BCVA) of 6/6 in each eye monocularly was mandatory to ensure reliable endpoints.

Exclusion criteria included any prior ocular surgery; keratoconus or corneal ectasia; corneal opacity; lens opacity of grade ≥1; posterior-segment pathology affecting refraction; narrow anterior chamber angles; congenital or acquired anterior-segment anomalies; chronic ocular inflammation; prior treatment for amblyopia or strabismus; and incomplete clinical records.

**Figure 1:**
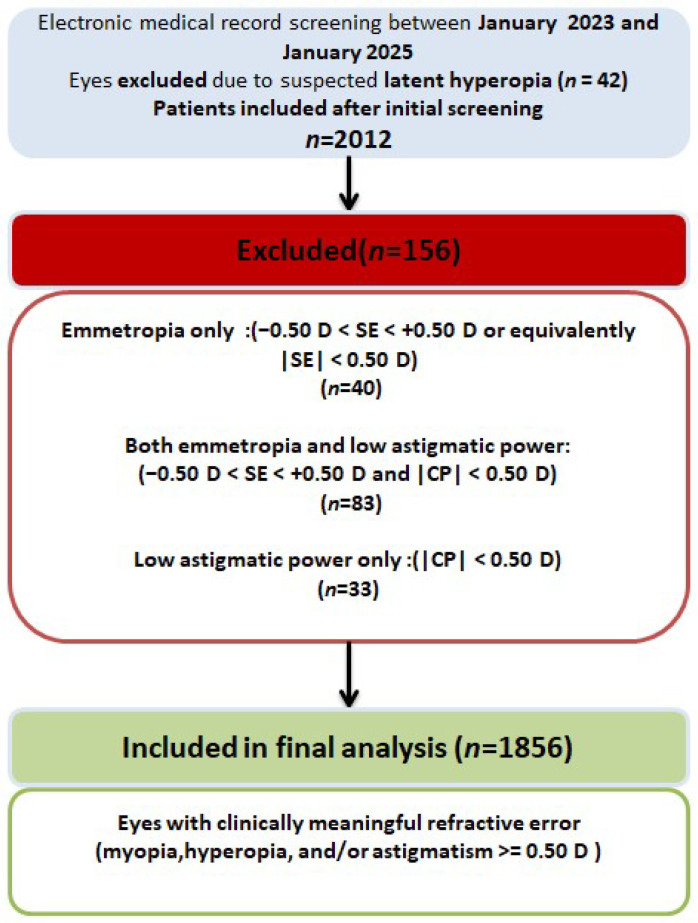
Study flow diagram showing patient selection and exclusion criteria.

Following patient-level eligibility screening, 1006 patients (2012 eyes) met the initial inclusion criteria. Subsequently, an eye-level filtering step was applied based on predefined refractive-error thresholds (see Section 2.4). A total of 156 eyes were excluded at this stage, yielding a final dataset of 1856 eyes for machine-learning model development (see Figure 1). All participants were recruited from a single tertiary referral center in Türkiye, and the study population was predominantly Middle Eastern/Caucasian.

### 2.2. Objective and Subjective Refraction Procedures

Objective measurements were obtained using an automated kerato-refractometer (KR-1; Topcon, Tokyo, Japan) under standardized low-illumination conditions (~80 lux), with internal fogging to reduce accommodation and monocular fixation on a 6 m high-contrast target. Subsequent to the initial acquisition, three consecutive readings were obtained and averaged. Measurements deviating by >±1.00 D triggered a repeat acquisition. Automatic centration and pupil alignment routines ensured consistency. The autorefractor was operated in accordance with the manufacturer’s standard maintenance and calibration protocols. All measurements were obtained under standardized conditions by two experienced ophthalmologists (O.C. and G.O.) to ensure procedural consistency and minimize operator-related variability.

Cycloplegia was not a standard procedure for preserving a realistic routine workflow. Eyes requiring cycloplegia for suspected latent hyperopia were excluded from the study.

Subjective refraction was performed by two experienced ophthalmologists (O.C. and G.O.) using a phoropter and Snellen chart positioned at 6 m (~300 lux). Autorefraction served as the initial procedure, followed by fogging and maximum-plus refinement to optimize visual acuity. Duochrome balancing was applied to confirm focal neutrality. The refinement of astigmatism entailed Jackson cross-cylinder testing (±0.25 D) with axis adjustments in 5-degree increments, followed by subsequent 2.5-degree refinement when a cylinder strength of ≥1.50 D was identified. To ensure the reliability of the results, additional reversal checks were employed. The process involved a sequence of steps, beginning with binocular balancing, followed by monocular optimization, and culminating in the recording of final values in minus-cylinder notation. Any discrepancy that exceeded ±0.25 D necessitated the confirmation of a senior reviewer.

### 2.3. Refractive Parameters and Classification

Refractive error was expressed using power-vector notation [18] whereby sphere, cylinder, and axis values were converted to spherical equivalent (SE), J0, and J45. The magnitude of the cylinder (CP) was derived from the J components. Detailed equations for forward and inverse transformations are provided in Appendix A to ensure reproducibility. Refractive categories were defined as follows: myopia (SE ≤ −0.50 D), hyperopia (SE ≥ +0.50 D), and emmetropia (|SE| < 0.50 D). The presence of astigmatism was defined as |CP| ≥ 0.50 D. Eyes failing to meet the established refractive-error criteria were excluded from model training.

### 2.4. Machine-Learning Model Development

Subjective SE, J0, and J45 served as target outputs for model prediction. A multi-output histogram gradient boosting regressor was implemented in Python (version 3.13.7) using Scikit-learn (version 1.7) and the KNIME Analytics Platform. The input features encompassed objective refraction (S, C, axis), derived vector components (SE, J0, J45), keratometry (K1, K2), and demographic variables (age, sex). Predicted vector outputs were subsequently transformed back into cylinder power and axis using inverse power-vector equations to enable clinical interpretation.

The development of machine learning (ML) models was based on data from both eyes for each subject. Hyperparameter optimization was conducted using a combination of grid search and patient-level GroupKFold cross-validation to prevent information leakage between fellow eyes. The search space was deliberately constrained to plausible ranges to control model complexity. Specifically, the depth of the trees was constrained to shallow configurations, with a maximum depth of 3. The histogram bin resolution was restricted to 64 or 128 bins, and the learning rates were maintained at low to moderate levels (0.02, 0.03, 0.05). Additional regularization was imposed through minimum samples per leaf (20 or 50) and L2 regularization penalties (5.0, 10.0). The maximum number of boosting iterations was set at 100 or 200, with early stopping determining the effective number of iterations. Detailed workflows and parameter configurations are provided in Appendix A and Appendix B.

The performance of the model was evaluated by means of the coefficient of determination (R^2^), the mean absolute error (MAE), and the root-mean-square error (RMSE). Given the circular nature of the astigmatic axis, angular residuals were wrapped within the 180° periodicity by computing the minimum angular difference (|Δθ| or 180° − |Δθ|). This was used to derive circular R^2^ and to fully eliminate boundary-related directional bias at the 0°/180° transition. In order to assess whether nonlinear modeling provided performance gains in addition to linear regression, additional gradient boosting models were evaluated within the training dataset for comparative purposes. This comparison was used solely to inform model selection; only the selected histogram-based gradient boosting model was subsequently evaluated on the independent held-out test dataset. The model reporting was conducted in accordance with the TRIPOD-AI checklist.

### 2.5. Model Performance Metrics

Model performance was evaluated on an independent, temporally separated held-out test dataset comprising patients recruited during a later period, with no patient overlap with the training data. The test dataset was not accessed during hyperparameter tuning or model development and was used exclusively for final performance evaluation. The prediction performance for spherical and cylindrical components was evaluated using the coefficient of determination (R^2^), mean absolute error (MAE), mean squared error (MSE), and root mean squared error (RMSE), all reported in diopters (D). Given the circular nature of astigmatic axis data, angular residuals were wrapped within the 180° periodicity by computing the minimum angular difference (|Δθ| or 180° − |Δθ|). The performance of the Axis prediction was consequently evaluated using circular correlation (ρ), mean and median absolute angular error, and clinically relevant success rates (≤5°, ≤10°, and ≤15°), reported in degrees (°).

### 2.6. Statistical Analysis

Statistical analyses were performed using Python (version 3.13.7) with the SciPy, Statsmodels (version 0.14.4), and Scikit-learn libraries. Continuous variables were summarized as mean ± standard deviation (SD) or median (interquartile range), depending on the distribution of the data. The assessment of normality was conducted through the implementation of the Shapiro–Wilk test and a Q–Q plot inspection. Although both eyes were utilized for machine-learning development, only right eyes were incorporated into inferential statistical analyses to circumvent the potential influence of correlated-eye bias. The prediction outcomes were dichotomized into two categories: well-predicted and poorly predicted eyes. These categories were determined using two predefined thresholds:

Primary definition: |ΔS| ≤ 0.50 D, |ΔC| ≤ 0.50 D, |ΔAxis| ≤ 10°;

Strict definition (sensitivity analysis): |ΔS| ≤ 0.25 D, |ΔC| ≤ 0.25 D, |ΔAxis| ≤ 5°.

Subsequently, group comparisons were conducted using Mann–Whitney U tests or independent-samples *t*-tests for continuous variables and chi-square or Fisher’s exact tests for categorical variables, as appropriate. The quantification of effect sizes was conducted by employing the epsilon-squared (ε^2^) metric for non-parametric comparisons. To identify independent predictors of poor prediction, multivariable logistic regression was performed using only objective pre-refraction variables (age, sex, objective SE, cylindrical magnitude, axis, and keratometry) to avoid circularity with the reference standard. Odds ratios (ORs) with 95% confidence intervals (CIs) were reported. A two-sided *p*-value less than 0.05 was considered statistically significant.

## 3. Results

### 3.1. Dataset Characteristics

A total of 1006 patients (2012 eyes) were initially assessed for eligibility. Following the application of predefined clinical and refractive exclusion criteria, 1856 eyes were selected for the development of a machine-learning model. The study population comprised 516 female patients (51.3%), exhibiting a broad age distribution that reflects routine clinical practice.

For model evaluation, an independent held-out test dataset comprising 214 eyes was analyzed separately. The refractive characteristics of both the training and independent test datasets, based on objective and subjective refraction, are summarized in Table 1.

Across the datasets examined, myopia was identified as the most prevalent refractive category, followed by hyperopia and emmetropia. The majority of eyes exhibited clinically significant astigmatism (|C| ≥ 0.50 D), with comparable distributions observed between the training and test datasets. The keratometric parameters (K1 and K2), derived from objective measurements, exhibited analogous central tendencies across datasets, thereby substantiating the comparability of corneal profiles. Objective refraction parameters are reported to characterize the input feature distribution of the AI model, whereas subjective refraction values represent the reference standard used for performance evaluation.

### 3.2. Model Performance

#### 3.2.1. Regression Performance

The regression performance of the proposed model was evaluated on an independent test dataset to assess performance beyond the training data. The accuracy of predictions for spherical and cylindrical components was measured using conventional regression metrics. However, due to the periodic nature of angular data, the performance of axis prediction was assessed using circular statistics.

In the context of spherical power prediction, the histogram gradient boosting model demonstrated a high explained variance (R^2^ = 0.987) with a mean absolute error (MAE) of 0.126 D. A comparable level of performance was observed in the cylindrical power prediction, with an R^2^ of 0.933 and an MAE of 0.137 D. This analysis also revealed negligible mean signed differences, indicating the absence of systematic directional bias.

The performance of the Axis prediction was evaluated using circular correlation and angular error metrics, as opposed to linear regression statistics. The model exhibited a robust circular correlation (ρ = 0.898), accompanied by a mean absolute angular error of 4.65° and a median absolute angular error of 0.96°. The clinical relevance of the success rates is further underscored by the fact that 91.9% of eyes were predicted with a deviation within ±5°, 93.1% within ±10°, and 93.6% within ±15°.

In order to assess whether the observed performance gains were attributable to nonlinear modeling rather than linear effects alone, a comparative evaluation was conducted between multivariable linear regression and gradient boosting approaches within the training dataset using identical input features. As demonstrated in Table 2, nonlinear gradient boosting models exhibited consistently improved performance across spherical, cylindrical, and axis predictions when compared with linear regression, as indicated by reduced prediction errors and enhanced agreement metrics. These findings support the presence of clinically meaningful nonlinearity and therefore led to the selection of the histogram-based gradient boosting model for subsequent independent test set evaluation.

#### 3.2.2. Overall and Stratified Prediction Performance in the Independent Test Dataset

The performance of the overall prediction model for spherical and cylindrical components, in addition to a stratified analysis of astigmatic axis prediction, is provided in Table 3. Across the independent test dataset, spherical and cylindrical predictions demonstrated high accuracy, with strong agreement between predicted and reference values. The cylindrical power prediction demonstrated an R^2^ of 0.903, accompanied by a mean absolute error of 0.152 D. In contrast, the spherical power prediction exhibited an R^2^ of 0.979, along with a mean absolute error of 0.157 D. A minimal mean signed difference was observed for both components, suggesting an absence of systematic over- or underestimation.

**Figure 2:**
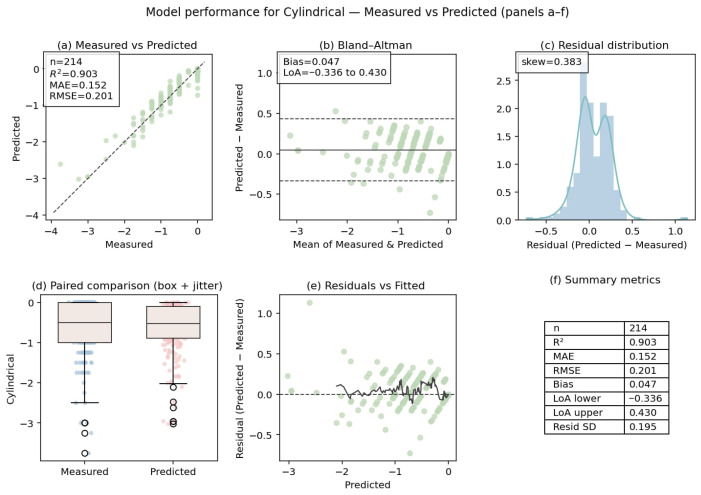
Agreement and error characteristics of cylindrical power prediction.

**Figure 3:**
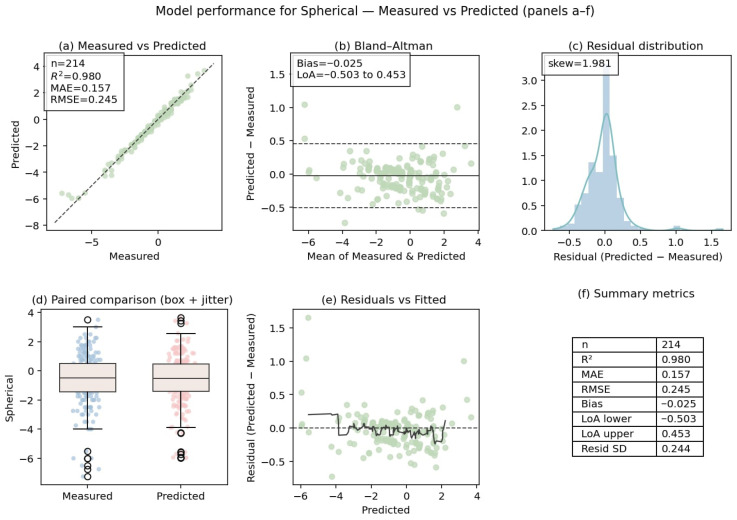
Agreement and error characteristics of spherical power prediction.

**Figure 4:**
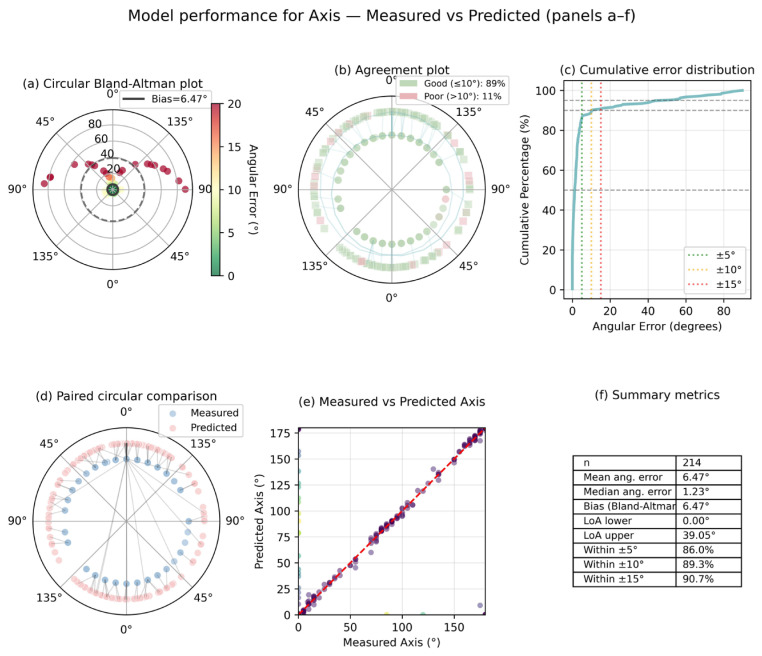
Circular performance of astigmatic axis prediction with clinically relevant error thresholds.

The performance of astigmatic axis prediction was evaluated using circular metrics and stratified by astigmatism type and cylinder magnitude. The overall axis prediction exhibited a strong circular correlation (ρ = 0.87), with a mean absolute angular error of 7.1° and a median absolute angular error of 1.23°. When the astigmatism types were stratified, the highest axis prediction accuracy was demonstrated by with-the-rule (WTR) astigmatism, exhibiting a circular correlation of 0.948 and a mean absolute angular error of 4.89°. Oblique astigmatism exhibited larger angular errors, with a mean absolute angular error of 8.09°. In contrast, against-the-rule (ATR) astigmatism demonstrated intermediate performance. The analysis of cylinder magnitude demonstrated variations in the accuracy of axis prediction across the groups. The mean absolute angular error was found to be 7.86° for eyes with cylinder magnitude <0.75 D, whereas eyes with 0.75–1.50 D and >1.50 D exhibited mean absolute angular errors of 4.73° and 5.61°, respectively. The corresponding success rates within predefined angular thresholds (≤5°, ≤10°, and ≤15°) are reported in Table 3 and Figure 2, Figure 3 and Figure 4.

#### 3.2.3. Classification Performance

**Figure 5:**
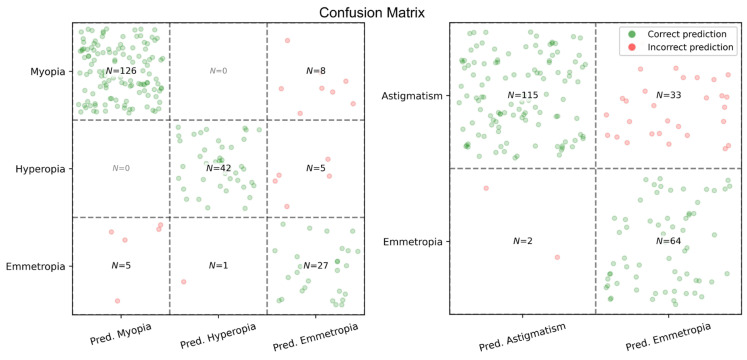
Classification performance for refractive status and astigmatism prediction.

The performance of the model in categorically classifying the refractive status is summarized in Table 4 and Figure 5. The classification accuracy was evaluated separately for three-class refractive status (myopia, hyperopia, emmetropia) and for binary astigmatism detection. The model demonstrated an overall accuracy of 91.1% for the three-class refractive status classification. The classification of myopia was determined by high recall (0.94) and precision (0.96), yielding an F-measure of 0.95. The hyperopia classification exhibited a recall of 0.89 and a precision of 0.98, resulting in an F-measure of 0.93.

The binary classification of clinically significant astigmatism (|C| ≥ 0.50 D) yielded an overall accuracy of 83.6%. The study identified astigmatism with a recall of 0.78 and a precision of 0.98, corresponding to an F-measure of 0.87. The emmetropic (non-astigmatic) class demonstrated a recall of 0.97, a precision of 0.66, and an F-measure of 0.79.

### 3.3. Clinical Factors Associated with Prediction Failure

Failure analyses were performed in the independent test dataset using right eyes only (*n* = 107) to avoid inter-eye correlation. Prediction outcomes were categorized as well-predicted or poorly predicted using two predefined tolerance thresholds: a primary definition (|ΔS| ≤ 0.50 D, |ΔC| ≤ 0.50 D, |ΔAxis| ≤ 10°) and a strict definition (|ΔS| ≤ 0.25 D, |ΔC| ≤ 0.25 D, |ΔAxis| ≤ 5°).

#### 3.3.1. Univariate Comparisons

According to the primary definition, 90 eyes were classified as well-predicted, while 17 eyes were classified as poorly predicted. The demographic, refractive, and keratometric characteristics of the two groups are compared and summarized in Table 5. No statistically significant differences were observed between groups for age, objective spherical power, objective cylindrical power, or objective axis. A comparison of the keratometric parameters revealed significant disparities between the study groups, with K2 values demonstrating higher levels in eyes that were poorly predicted.

According to the strict definition, 62 eyes were classified as “well-predicted,” while 45 eyes were classified as “poorly predicted.” Group comparisons under this stricter threshold also demonstrated differences in keratometric parameters, particularly for K2. However, other refractive and demographic variables exhibited overlapping distributions between groups. The effect sizes for non-parametric comparisons are documented in Table 5.

#### 3.3.2. Multivariable Logistic Regression Analysis

To identify independent predictors of poor prediction, a multivariable logistic regression model was constructed using the primary error definition as the outcome. Only objective pre-refraction variables were included as predictors. The results of the regression analysis are presented in Table 6.

In the multivariable model, steeper keratometry (K2) was associated with an increased likelihood of poor prediction (odds ratio [OR] = 7.25; 95% confidence interval [CI], 1.62–32.46; *p* = 0.010). Objective cylindrical power was also associated with poor prediction (OR = 2.79; 95% CI, 1.87–8.94; *p* = 0.032). In addition, the adjusted model revealed that factors such as age, sex, objective spherical power, objective axis, and K1 were not significantly associated with prediction outcome.

## 4. Discussion

The present study demonstrates that a machine-learning model trained exclusively on routinely available, non-cycloplegic autorefraction and keratometric data can achieve clinically meaningful prediction of subjective refraction. Notably, the model demonstrated the capacity to predict not only spherical equivalent, but also cylindrical power and astigmatic axis, utilizing inputs that are a component of standard ophthalmic examinations. By emphasizing the utilization of readily available measurements as opposed to the employment of specialized imaging or biometric devices, the present study aims to overcome a significant impediment to the practical implementation of artificial intelligence-assisted refraction in real-world settings.

A comparative analysis of model performance across various refractive components revealed that prediction accuracy was most pronounced for spherical and cylindrical components. In the independent test dataset, both parameters exhibited low absolute errors and negligible mean signed differences, indicating the absence of systematic overcorrection or undercorrection. From a clinical perspective, this lack of directional bias is particularly important, as even minor systematic shifts in spherical or cylindrical power can result in patient discomfort or diminished visual satisfaction despite relatively low absolute error values.

These findings are consistent with, and in some cases comparable to, previous machine-learning-based refractive prediction studies that incorporated biometric measurements such as axial length, anterior corneal surface metrics, or wavefront aberrometry [6,7,8,12]. However, many of these approaches depend on advanced instrumentation that is not routinely available in primary care or high-volume ophthalmology clinics. In contrast, the present study demonstrates that comparable predictive performance for spherical and cylindrical components can be achieved using only standard autorefractor and keratometry data. This distinction is clinically significant because it prioritizes scalability and feasibility over maximal theoretical accuracy. Together, these findings indicate that clinically acceptable refractive prediction can be achieved without reliance on specialized imaging or biometric devices.

When evaluated in comparison with multivariable linear regression, gradient boosting approaches exhibited a consistent enhancement in predictive performance across spherical, cylindrical, and axis components within the training dataset. This finding suggests that nonlinear modeling may more effectively capture the intricate relationships underlying refractive outcomes than simple linear correction of autorefractor bias. The consistent observation of these performance gains across various nonlinear models lends support to the hypothesis of the presence of a clinically meaningful nonlinear structure, rather than merely bias adjustment. Despite the high explained variance for spherical and cylindrical components, which necessitates meticulous interpretation, model complexity was explicitly constrained through shallow tree depth, regularization, and patient-level grouped cross-validation, thereby reducing the likelihood of overfitting. Given the comparable performance across gradient boosting approaches, histogram-based gradient boosting was selected due to its greater stability in the presence of measurement noise inherent in routine autorefraction and keratometry data. The selection of this approach placed a priority on stability and reliability in real-world clinical use, rather than on marginal gains in predictive accuracy [6,7,12].

While the model demonstrated strong performance for spherical and cylindrical components, the prediction of astigmatic axis proved to be inherently more challenging. This discrepancy is not unexpected and reflects fundamental properties of axis measurements rather than a limitation of the modeling approach. Unlike spherical and cylindrical power, the astigmatic axis is a circular variable with 180° periodicity, for which conventional linear error metrics are inappropriate and may substantially misrepresent true prediction error, particularly near the 0°/180° boundary. Accordingly, axis performance in the present study was evaluated using circular correlation and angular error–based metrics with residuals wrapped within the 180° periodicity, ensuring mathematically and clinically meaningful interpretation [18]. Despite appropriate circular handling, axis prediction accuracy remained lower than that of spherical and cylindrical components. This finding is consistent with prior refractive modeling studies, which have reported greater intrinsic variability in axis estimation compared with spherical and cylindrical power, particularly in eyes with low cylindrical magnitude [6,7,12]. In such cases, small absolute changes in cylinder magnitude may result in disproportionately large angular deviations despite minimal optical or clinical impact. Accordingly, absolute angular error alone may overstate the clinical relevance of axis disagreement when cylinder power is low. This consideration underscores the importance of stratified analyses that contextualize axis error by cylinder magnitude and astigmatism type.

Stratified analyses further demonstrated that axis prediction accuracy was substantially higher in eyes with moderate-to-high astigmatism and with-the-rule or against-the-rule configurations, whereas larger angular deviations were predominantly confined to low-cylinder or oblique astigmatism. Similar patterns have been reported in previous machine-learning–based refractive studies, supporting the interpretation that reduced axis performance is largely confined to refractive scenarios in which axis precision is both more variable and less clinically consequential [6,12].

Beyond aggregate accuracy metrics, we further examined the clinical conditions under which prediction failure is most likely to occur. Rather than relying solely on aggregate accuracy measures, we investigated which eyes were more likely to fall outside clinically acceptable error thresholds. A pragmatic primary definition that reflects routine clinical tolerance (|ΔS| ≤ 0.50 D, |ΔC| ≤ 0.50 D, |ΔAxis| ≤ 10°) was utilized in a multivariable logistic regression analysis. This analysis identified keratometric parameters, particularly steeper K2 values, and objective cylindrical power as independent predictors of poorer prediction performance. Age, sex, and spherical power were not independently associated with prediction failure after adjustment. Taken together, these findings indicate that corneal steepness and increasing cylindrical complexity may introduce additional sources of variability that are not fully captured by routine autorefractor measurements. Importantly, although low cylinder magnitude was associated with larger angular axis errors in univariate analyses, higher cylindrical power emerged as a determinant of overall prediction failure in multivariable modeling, reflecting the combined contribution of sphere, cylinder, and axis errors rather than axis instability alone. By identifying predictors of reduced accuracy, the present analysis provides clinically actionable insight into the conditions under which AI-assisted refraction is most likely to perform reliably.

From a practical standpoint, the findings support a hybrid implementation strategy in which machine-learning–assisted refraction functions as a decision-support tool rather than a replacement for subjective refraction. In cases of definite refractive errors and moderate-to-high astigmatism, the model provides precise initial values that have the potential to enhance the efficiency of clinical procedures. Conversely, eyes exhibiting low cylinder magnitude, oblique astigmatism, or steeper corneal profiles can be readily identified as requiring full subjective refinement. This approach aligns with current recommendations for the responsible and trustworthy clinical deployment of artificial intelligence in eye care [19,20].

The current model provides point estimates only and was intentionally evaluated under this constraint to reflect routine autorefractor output. In this context, it is important to note that model performance was evaluated at the population level, without explicit modeling of individual-level prediction uncertainty. Uncertainty-aware approaches, including prediction intervals, quantile regression, and confidence scoring, are recognized as important next steps for safe clinical implementation and are prioritized for future prospective studies.

Several limitations of the present study must be acknowledged. First, all measurements were obtained using a single autorefractor model (Topcon KR-1). Consequently, the model’s predictive performance may partly reflect learned instrument-specific characteristics rather than purely device-independent refractive patterns. Autorefractors differ across manufacturers in optical design, internal algorithms, keratometric assumptions, and calibration protocols, which may limit the direct transferability of the present findings across platforms. Accordingly, deployment on alternative autorefractors (e.g., Nidek or Zeiss) would likely require device-specific recalibration or modest local fine-tuning to correct for systematic offsets. Second, the study population was derived from a single tertiary referral center and was predominantly Middle Eastern/Caucasian; therefore, external validation in ethnically diverse cohorts is warranted to confirm population-level generalizability. However, model performance was evaluated using a temporally separated independent test cohort recruited during a later period, which partially mitigates concerns regarding overfitting and temporal instability of model performance.

Moreover, the routine non-use of cycloplegia may have limited the detection of latent hyperopia, particularly in younger individuals, although this reflects common real-world clinical practice. Furthermore, the requirement of best-corrected visual acuity of 6/6 was applied to ensure a reliable reference standard for subjective refraction. However, this criterion may limit the applicability of the findings to patients with media opacities or reduced visual acuity. Finally, the model did not incorporate biometric predictors such as axial length or higher-order aberrations, which have been shown to improve refractive estimation in borderline or complex cases. In addition, predictors of reduced prediction accuracy were also examined retrospectively, and the number of poorly predicted eyes was limited; accordingly, the multivariable logistic regression analysis should be interpreted as exploratory rather than confirmatory.

From a clinical perspective, the integration of AI-assisted refraction as a decision-support tool within routine ophthalmic practice is likely to be more effective than its use as a direct replacement for subjective refraction. In high-volume clinical settings, the proposed model could provide an initial refractive estimate prior to or during subjective refraction, potentially reducing examination time, facilitating patient flow, and preserving clinician oversight. Because the model relies exclusively on routinely acquired autorefraction and keratometry measurements, its implementation would not require additional devices or procedural adjustments. Importantly, such integration may allow clinicians to focus subjective refinement on cases with higher predicted uncertainty, thereby improving efficiency without compromising visual outcomes or patient satisfaction. However, the clinical utility of this approach has yet to be formally quantified in prospective settings, and broader clinical adoption should therefore proceed through cautious, stepwise validation rather than assumed universal deployment.

## 5. Conclusions

Our study demonstrates that a machine-learning model based on routine, non-cycloplegic autorefractor and keratometric data can accurately estimate subjective refraction. The model demonstrated high levels of agreement with respect to spherical and cylindrical components. The model’s performance was further demonstrated to be clinically interpretable for astigmatic axis prediction when evaluated with circular error metrics. However, the accuracy of these predictions varied according to refractive profiles, with reduced performance primarily observed in cases of low cylinder magnitude, oblique astigmatism, or steeper corneal curvature. Overall, these findings support the use of artificial intelligence as a complementary decision-support tool that assists, rather than replaces, conventional subjective refraction.

## Figures and Tables

**Table 1:** Refractive characteristics of the training and test datasets based on objective and subjective refraction.

	Training Data		Test Data	
	Objective Refraction	Subjective Refraction	Objective Refraction	Subjective Refraction
**Spherical equivalent (D), mean ± SD**	−1.14 ± 2.01	−1.06 ± 1.87	−0.99 ± 1.94	−0.92 ± 1.81
**Myopia, *n* (%)**	1252 (68%)	1250 (67%)	159 (74%)	134 (63%)
**Emmetropia, *n* (%)**	-	258 (14%)	-	33 (15%)
**Hyperopia, *n* (%)**	597 (32%)	348 (19%)	54 (26%)	47 (22%)
**|C| (D), mean ± SD**	0.94 ± 0.75	0.70 ± 0.69	0.84 ± 0.70	0.68 ± 0.65
**Clinically significant astigmatism (|C| ≥ 0.50 D), *n* (%)**	1291 (70%)	1063 (57%)	157 (73%)	148 (69%)
**K1 (D)**	43.15 ± 1.26	-	43.31 ± 1.04	-
**K2 (D)**	43.87 ± 1.25	-	44.19 ± 0.99	-
**Age (years)**	36.47 ± 18.44		38.6 ± 18.60	

D = diopters; SD = standard deviation. C denotes cylinder power; |C| represents the absolute value (magnitude) of the cylindrical refractive error. Objective refraction values are presented to describe the input distribution of the AI model, whereas subjective refraction represents the reference standard used for model evaluation. Myopia was defined as SE ≤ −0.50 D, emmetropia as −0.50 D < SE < +0.50 D, and hyperopia as SE ≥ +0.50 D. K1 and K2 represent flat and steep keratometric powers derived from objective measurements.

**Table 2:** Performance comparison of linear regression and gradient boosting models for axis, cylindrical, and spherical prediction within the training dataset.

	Cylindrical			Spherical				Axis		
	LR	XGB	HGBT	LR	XGB	HGBT		LR	XGB	HGBT
**R^2^**	0.829	0.927	0.933	0.96	0.989	0.987	**Circular Correlation (Rho)**	0.777	0.888	0.898
**Mean Absolute Error (D)**	0.184	0.148	0.137	0.167	0.128	0.126	**Mean Absolute Error (°)**	12.47	5.33	4.65
**Mean Squared Error (D)**	0.064	0.035	0.032	0.067	0.037	0.041	**Median Absolute Error (°)**	1.57	0.97	0.96
**Root Mean Squared Error (D)**	0.253	0.188	0.18	0.26	0.194	0.203	**Success Rate (≤5°)**	74.30%	89.80%	91.90%
**Mean Signed Difference (D)**	0.000	0.002	0.01	0.000	0.000	−0.006	**Success Rate (≤10°)**	77.50%	92.80%	93.10%
							**Success Rate (≤15°)**	79.90%	93.60%	93.60%

LR = linear regression; HGBT = histogram-based gradient boosting trees; XGB = eXtreme Gradient Boosting. For axis prediction, performance was evaluated using circular correlation and angular error metrics due to the circular nature of astigmatic axis data. Circular correlation (ρ) was computed using circular–circular correlation adapted for angular variables. Angular residuals were calculated with circular correction by taking the minimum angular difference within 180°. Angular errors are reported in degrees (°). Success rates represent the proportion of eyes with absolute angular error within the specified thresholds (≤5°, ≤10°, and ≤15°). For cylindrical and spherical prediction, performance was evaluated using linear regression metrics, including R^2^, mean absolute error, mean squared error, root mean squared error, and mean signed difference. Cylindrical and spherical errors are reported in diopters (D).

**Table 3:** Overall prediction performance for spherical and cylindrical components and stratified astigmatic axis prediction analysis in the independent test dataset.

	**Predicted Variable**	**R^2^**	**Mean Absolute Error**	**Mean Squared Error**	**Root Mean Squared Error**	**Mean Signed Difference**	
**All**	**Cylindrical**	0.903	0.152	0.04	0.201	0.047	-
	**Spherical**	0.979	0.157	0.059	0.244	−0.025	-
	**Predicted Variable**	**Circular Correlation (Rho)**	**Mean Absolute Angular Error (°)**	**Median Absolute Angular Error (°)**	**Success Rate (** **≤5°)**	**Success Rate (** **≤10°)**	**Success Rate (** **≤15°)**
**All**	**Axis**	0.87	7.1	1.23	84%	89.30%	90.70%
**Oblique**	**Axis**	0.798	8.09	1.52	82%	87%	88.40%
**WTR**	**Axis**	0.948	4.89	1.99	94%	95.40%	96.30%
**ATR**	**Axis**	0.928	5.55	1.12	93%	98.70%	100%
**<0.75 D**	**Axis**	0.784	7.86	1.28	87%	91.80%	93.70%
**0.75–1.50 D**	**Axis**	0.94	4.73	1.3	95%	100%	100%
**>1.50 D**	**Axis**	0.929	5.61	1.68	96%	100%	100%

Prediction performance for spherical and cylindrical components was evaluated using conventional regression metrics and is reported in diopters (D). Astigmatic axis prediction performance was evaluated using circular correlation and angular error metrics due to the circular nature of axis data, with angular errors reported in degrees (°). Stratified analyses for axis prediction were performed by astigmatism type and cylinder magnitude. Success rates represent the proportion of eyes with absolute angular error within predefined clinical thresholds.

**Table 4:** Categorical classification performance for refractive status.

	**Pred. Myopia**	**Pred. Hyperopia**	**Pred. Emmetropia**	**Recall**	**Precision**	**F-Measure**	**Overall Accuracy**
**Myopia**	126	0	8	0.94	0.96	0.95	**91.1%**
**Hyperopia**	0	42	5	0.89	0.98	0.93	
**Emmetropia**	5	1	27	0.82	0.68	0.74	
	**Pred. Astigmatism**	**Pred. Emmetropia**		**Recall**	**Precision**	**F-Measure**	**Overall Accuracy**
**Astigmatism**	115	33		0.78	0.98	0.87	**83.6%**
**Emmetropia**	2	64		0.97	0.66	0.79	

Classification metrics were computed in a one-vs-rest manner for each class.

**Table 5:** Characteristics of well- and poorly predicted eyes using primary and strict definitions.

**Primary Definition (Sphere/Cylinder ≤ 0.50 D, Axis ≤ 10°)**								
	***N***	**Mean** **±** **SD**	**Median (Min–Max)**	***N***	**Mean** **±** **SD**	**Median (Min–Max)**	***p***	**Effect Size (ε^2^)**
	**Well-Predicted**			**Poorly Predicted**				
**Age**	90	38.42 ± 19.04	37.00 (8.00/77.00)	17	39.53 ± 16.26	39.00 (14.00/65.00)	0.717	0.00
**Spherical (Autorefraction)**	90	−0.54 ± 1.75	−0.50 (−6.50/3.75)	17	−0.71 ± 2.40	−0.75 (−7.25/3.25)	0.959	0.00
**Cylindrical (Autorefraction)**	90	−0.83 ± 0.68	−0.75 (−3.75/0.00)	17	−0.82 ± 1.03	−0.25 (−4.25/−0.25)	0.245	0.003
**Axis (Autorefraction)**	90	82.40 ± 66.59	80.00 (0.00/180.00)	17	92.35 ± 67.54	103.00 (1.00/180.00)	0.440	0.00
**K1**	90	43.19 ± 1.01	43.00 (41.25/45.75)	17	43.69 ± 1.00	43.75 (41.50/45.00)	**0.046**	0.028
**K2**	90	44.04 ± 0.98	44.00 (41.00/46.50)	17	44.78 ± 0.71	44.75 (43.25/45.75)	**0.004**	0.077
**Spherical (Subjective)**	90	−0.53 ± 1.63	−0.50 (−6.00/3.50)	17	−0.79 ± 2.20	−0.75 (−7.25/2.25)	0.814	0.00
**Cylindrical (Subjective)**	90	−0.69 ± 0.59	−0.50 (−3.25/0.00)	17	−0.53 ± 1.02	0.00 (−3.75/0.00)	**0.016**	0.045
**Axis (°) (Subjective)**	90	73.66 ± 67.55	75.00 (0.00/180.00)	17	38.82 ± 71.84	0.00 (0.00/180.00)	**0.013**	0.05
	***N***			***N***				
**Gender (F/M)**	90 (58%/42%)			17 (70%/30%)			0.323 *	
**Strict Definition (Sphere/Cylinder ≤ 0.25 D, Axis ≤ 5°)**								
	***N***	**Mean** **±** **SD**	**Median (Min–Max)**	***N***	**Mean** **±** **SD**	**Median (Min–Max)**	***p***	**Effect Size (ε^2^)**
	**Well-Predicted**			**Poorly Predicted**				
**Age**	62	35.87 ± 18.42	33.00 (8.00/77.00)	45	42.36 ± 18.30	45.00 (12.00/75.00)	0.082	0.019
**Spherical (Autorefraction)**	62	−0.66 ± 1.54	−0.75 (−6.50/3.75)	45	−0.44 ± 2.23	0.00 (−7.25/3.25)	0.175	0.008
**Cylindrical (Autorefraction)**	62	−0.75 ± 0.70	−0.62 (−3.75/0.00)	45	−0.94 ± 0.79	−0.75 (−4.25/0.00)	0.180	0.008
**Axis (Autorefraction)**	62	75.77 ± 69.98	62.50 (0.00/179.00)	45	95.29 ± 60.37	99.00 (0.00/180.00)	0.092	0.017
**K1**	62	43.19 ± 1.12	43.00 (41.25/45.75)	45	43.38 ± 0.88	43.25 (41.50/45.00)	0.190	0.007
**K2**	62	43.94 ± 1.08	43.75 (41.00/46.50)	45	44.45 ± 0.73	44.50 (43.25/45.75)	**0.004**	0.069
**Spherical (Subjective)**	62	−0.65 ± 1.41	−0.62 (−6.00/3.50)	45	−0.47 ± 2.09	0.00 (−7.25/2.50)	0.170	0.008
**Cylindrical (Subjective)**	62	−0.61 ± 0.62	−0.50 (−3.25/0.00)	45	−0.74 ± 0.75	−0.50 (−3.75/0.00)	0.355	0.00
**Axis (°) (Subjective)**	62	65.23 ± 69.99	35.00 (0.00/180.00)	45	72.11 ± 68.41	80.00 (0.00/180.00)	0.646	0.00
	***N***			***N***				
**Gender (F/M)**	62 (55%/45%)			45 (66%/34%)			0.218 *	

D = diopters. Well- and poorly predicted eyes were defined using two different error thresholds. The primary definition was based on clinically pragmatic criteria (|ΔS| ≤ 0.50 D, |ΔC| ≤ 0.50 D, and |ΔAxis| ≤ 10°), which was used for the multivariable logistic regression analysis. A stricter definition (|ΔS| ≤ 0.25 D, |ΔC| ≤ 0.25 D, and |ΔAxis| ≤ 5°) was additionally evaluated as a sensitivity analysis. Continuous variables are presented as mean ± standard deviation and median (minimum–maximum), as appropriate. C denotes cylindrical power; |C| represents the absolute value (magnitude) of the cylindrical refractive error. Axis values are reported in degrees (°) and are presented for descriptive purposes only; inferential statistical testing was not performed for axis due to the circular nature of angular data. Statistically significant values were indicated in bold font. * The chi-square test was used to generate a *p* value.

**Table 6:** Multivariable logistic regression analysis of predictors of poor prediction using the primary error definition (|ΔS| ≤ 0.50 D, |ΔC| ≤ 0.50 D, |ΔAxis| ≤ 10°).

	*p* Value	Odds Ratio	95% CI (Lower–Upper)	
**Age**	0.587	0.990	0.956	1.026
**Gender(Female)**	0.780	1.193	0.346	4.113
**Spherical (Autorefraction)**	0.325	1.202	0.834	1.732
**Cylindrical (Autorefraction)**	**0.032**	2.792	1.872	8.935
**Axis (Autorefraction)**	0.576	1.003	0.994	1.011
**K1 (D)**	0.097	0.339	0.095	1.214
**K2 (D)**	**0.010**	7.251	1.620	32.461

Odds ratios are reported per 1-unit increase in each predictor. Only objective pre-refraction variables were included in the multivariable model to avoid circularity with the reference standard. D = diopters; CI = confidence interval; K1 and K2 denote flat and steep keratometric powers, respectively. Statistically significant values were indicated in bold font.

## Data Availability

Due to ethical and legal privacy restrictions related to patient medical records, the clinical datasets used and analyzed in this study cannot be publicly shared. De-identified data may be made available from the corresponding author upon reasonable request and subject to approval by the institutional ethics committee.

## Appendix A

Detailed mathematical formulations for power-vector conversion (SE, J0, J45) and inverse reconstruction into spherical power, cylinder power, and axis are presented in Appendix A.

$$SE=S+\frac{C}{2}$$

$$a=A\times \frac{2\pi }{180}$$

$$J_{0}=-\frac{1}{2}\times C\times \cos\;(2\;a)$$

$$J_{45}=-\frac{1}{2}\times C\times \sin\;(2\;a)$$

$$C=-2\times \sqrt{{\left(J_{0}\right)}^{2}+{\left(J_{45}\right)}^{2}}$$

$$a=\left(\frac{1}{2}\right)\times arctan2\;\left(J_{45},J_{0}\right)$$

## Appendix B

The full machine-learning workflow, including data preprocessing, feature generation, model architecture, and training/evaluation pipeline, is illustrated schematically in Appendix B.

## Appendix C

Machine learning model inputs are illustrated schematically in Appendix C.

**Machine Learning Model Inputs**		
**Variable Name**	**Definition/Calculation**	**Role in Model**
**Patient_id**	Patient identifier	Not used in training (only for tracking)
**S_auto**	Sphere measured by autorefractor (D)	Raw input
**C_auto**	Cylinder measured by autorefractor (D)	Raw input
**Axis_auto**	Cylinder axis measured by autorefractor (°)	Raw input
**M_auto**	S_auto + C_auto/2 (spherical equivalent, SE)	Power vector input
**J0_auto**	−(C_auto/2) × cos (2 × Axis_auto)	Power vector input (horizontal/vertical astigmatism)
**J45_auto**	−(C_auto/2) × sin (2 × Axis_auto)	Power vector input (oblique astigmatism)
**M_a2, J0_a2, J45_a2**	Squared terms of power vector components	Non-linear feature expansion
**M_aJ0_a, M_aJ45_a, J0_a*J45_a**	Interaction terms among power vectors	Feature interactions
**Cabs_a**	Absolute value of C_auto	C
**age**	Patient age (years)	Demographic input
**age2**	Squared age	Non-linear age effect
**sex_Female/Male/Other**	Dummy variables derived from sex	Demographic input
**K1, K2**	Keratometry values (D)	Corneal shape input
**Kmean**	(K1 + K2)/2	Mean corneal curvature
**Kdelta**	K1 − K2	K1 − K2
**Kratio**	K1/K2	Corneal asymmetry
**age Kmean, age Kdelta**	Interaction of age with corneal shape	Interaction features
**S_man**	Sphere measured manually by clinician (D)	Target (training only)
**C_man**	Cylinder measured manually by clinician (D)	Target (training only)
**Axis_man**	Cylinder axis measured manually by clinician (°)	Target (training only)
**M_true**	Power vector M from manual refraction	Target (training only)
**J0_true**	Power vector J0 from manual refraction	Target (training only)
**J45_true**	Power vector J45 from manual refraction	Target (training only)
**SE_true**	Manual spherical equivalent (S_man + C_man/2)	Target (training only)
**Cabs_true**	Absolute cylinder from manual refraction	Target (training only)
